# Selectivity of Migration and the Educational Disadvantages of Second-Generation Immigrants in Ten Host Societies

**DOI:** 10.1007/s10680-018-9484-2

**Published:** 2018-03-27

**Authors:** Herman G. van de Werfhorst, Anthony Heath

**Affiliations:** 1 Sociology, University of Amsterdam, PO Box 15508, 1001 NA Amsterdam, The Netherlands; 2 Sociology, Nuffield College, University of Oxford, Oxford, UK

**Keywords:** Ethnicity, Educational inequality, Migration, Selective migration, Integration policies

## Abstract

Selectivity of migration varies significantly between ethnic/origin country groups, and between the destination countries which these groups have migrated to. Yet, little comparative research has measured empirically how selective different migrant groups are in multiple destination countries, nor has research studied whether the selectivity of migration is related to the magnitude of ethnic inequalities among the children of migrants in Western societies. We present an empirical measure of educational selectivity of migrants from many different origin countries having migrated to ten different destination countries. We examine whether selective migration of a particular ethnic group in a particular destination country is related to the gap between their children’s and native children’s educational outcomes. We find that the disadvantage in educational outcomes between the second generation and their peers from majority populations is smaller for ethnic groups that are more positively selected in terms of educational attainment. We also find some evidence that the effect of selective migration is moderated by the integration policies or tracking arrangements in the educational system in the destination country.

## Introduction

Processes of first- and second-generation integration vary strongly between migrants of different origin countries and between the countries they have migrated to. One domain of integration concerns the educational attainment of the children of immigrants: are second-generation children of migrants disadvantaged in the education system compared to children of the majority population in the host country? Some groups, such as Asian migrants to the USA, are known to have exceptionally good performances compared to the majority populations, while other groups, such as children of the Mediterranean labor migrants to Europe of the 1960s–1970s, do much worse (Heath et al. 2008).

One potential source of these stark differences among immigrant groups across different countries has been hardly addressed in the comparative literature: the selectivity of the migrant population. A number of single-country studies have shown that emigrants are positively selected on educational attainment compared to the homestayers (e.g., Feliciano 2005a; Bertoli et al. 2013; Ichou 2014). Others have examined selectivity of migration for a large number of countries, but have not examined the consequences for the integration into the educational system of the children of immigrants (Brücker and Defoort 2009; Belot and Hatton 2012).

Given that parental education is one of the most important predictors of children’s educational attainment, it is likely that positive selection has implications for immigrants’ integration into the host society. Moreover, selectivity of migration may partially explain the significant differences in the educational outcomes across ethnic groups in various destination countries. Especially, second-generation children’s educational achievement and attainment may benefit from a community that is ‘positively selected’ in terms of education, over and above the positive effects generated by the educational attainment of parents. Ethnic communities are defined here as the *combination* of origin country and destination country (Van Tubergen et al. 2004). It is likely that positively selected communities generate particularly high aspirations for children. These aspirations may translate into more ambitious educational choices and achievements, as argued by Kao and Tienda (1995).

Furthermore, while selectivity of migration may contribute to our understanding of variations in the educational disadvantage of different immigrant groups, it also is plausible that its importance varies across destination countries. Positively selected migrant communities may be less disadvantaged compared to neutrally or negatively selected communities, but the magnitude of these differences are likely to be modified by destination country institutions. In some destination countries, low-educated communities may be at a greater disadvantage than in other countries, depending on the types of integration policies or the nature of the educational system.

In this paper, we take up the challenge to study these comparative research problems. We present an index of selective migration into ten Western host societies, across 34 distinct ethnic groups, totaling 81 ‘communities.’ We examine whether selectivity of migration is related to the extent to which second-generation groups are disadvantaged across various educational outcomes, in comparison with majority populations. We then examine whether the educational disadvantage of negatively selected migrant groups is particularly found in destination countries with unfavorable integration policies or strongly diversified educational systems. Doing so, our approach follows a research line that emphasizes that integration and assimilation of migrants (and their children) is a function of the migration context (Van Tubergen et al. 2004; Van Tubergen and Kalmijn 2005; Crul and Schneider 2010; Hillmert 2013). Integration is affected by characteristics of the destination country (such as migrant integration policies and educational systems) and of the community of migrant groups in a particular destination country. Moreover, we study whether destination country institutions have differential effects on different groups of migrants. These interaction effects between the selectivity of ethnic communities and destination country policies have not been studied before.

We use a harmonized dataset comprising the best available national datasets to study ethnic educational inequality, brought together for a large comparative project (Heath and Brinbaum 2014). For the ten destination countries, we harmonized existing data that cover three outcomes in various crucial stages of the secondary school career: (1) the performance in standardized test scores in the first stage of secondary education; (2) whether a vocational or academic route is chosen at upper secondary education; and (3) whether upper secondary education has been completed. Our data thus enable the study of multiple outcomes in the school career, instead of only test scores which are the major object of study in the existing literature. The study of multiple educational outcomes has been called for by Alba et al. (2011). We can control for parental education and occupation in order to find ‘net effects’ of ethnicity in comparison with ‘similar’ children of majority populations on all these outcomes. Moreover, a unique feature of our data is that we construct a ‘net difference index’ of selectivity of migration with regard to human capital for all ethnic groups in ten host countries and associate it with racial/ethnic educational inequality. While following the approach of Feliciano (2005a), our measure is unique because it covers multiple host societies instead of one, which enables us to study migrants in a multiple-origin multiple-destination design.

## Selective Migration in Context: Theory and Hypotheses on Second-Generation Immigrants’ Education

### Selective Migration

A number of studies have examined whether immigrant communities are positively or negatively selected in comparison with the non-migrants in the origin country (Borjas 1987; Bertoli et al. 2013; Chiswick 1999; Ichou 2014). Migrants are reported to be positively self-selected on educational attainment, motivation, ability, and effort, compared to the homestayers. Even if the (positive) returns to education are equal between origin and destination countries, economic theory would predict positive selection on education given a fixed amount of relocation costs for all migrants (Chiswick 1999). Yet, societal circumstances (of both the origin and destination countries) can have effects on the extent to which there is selectivity of migration. If the returns to human capital are higher in destination countries, selectivity will be stronger, and selectivity is stronger if there are wealth or income constraints (as, for instance, imposed by larger geographical distance or other obstacles between origin and destination) (Bertoli et al. 2013; Borjas 1987). In line with this economic model, Van Tubergen et al. (2004) indirectly studied selectivity of migration for each immigrant group in a great number of destination countries by comparing the level of income inequality between the destination and origin countries. A comparatively high level of inequality in the destination country would be indicative of positive selection on income-generating characteristics (skills, education), as the returns to human capital are higher in high-inequality countries.

Essential of this literature is that, both between and within destination countries, there is a wide variety of selectivity between migrant groups. Observed levels of selectivity of migrant groups in different host societies result from two distinct processes. First, some people may be more likely to migrate abroad than others (selectivity), and second, migrants choose countries based on the expected returns to their skills (sorting, Grogger and Hanson 2011). A wide variety in educational selectivity has been demonstrated for the USA (Feliciano 2005a). For almost all of the 32 immigrant communities that were studied, selection in terms of educational attainment was positive (except for Puerto Rico), but much less positive for central American immigrants to the USA than for Europeans and Asians. Factors associated with selectivity were geographical distance to the USA (more selectivity if the distance is larger) and the average level of education in the home country (less selectivity in more highly educated origin countries).

Selective migration can be expected to be associated with immigrant integration and assimilation. For the USA, it has been demonstrated that positive selection (indicated by low inequality in the origin country and larger geographical distance) was related to higher earnings of first- and second-generation migrants (Borjas 1993). Moreover, the integration outcomes are not restricted to migrants themselves, but can be transmitted to the next generation of children born in the destination country. Feliciano (2005b) found that college enrollment of second-generation students was higher for more strongly educationally selected communities. Moreover, differences in selectivity accounted for differences in college completion rates between ethnic groups, particularly for the high rates among Asian immigrants. Kao and Tienda (1995: 5) explained the high achievement of second-generation Asians by “their parents’ optimism about their socioeconomic prospects [which] leads youth to behave in ways that promote educational success.” Thus, selectively migrated groups are likely to have resources in their communities beyond parental educational levels, which are likely to help the structural integration of the second generation.

Much less is known about the relevance of migration selectivity on integration in cross-national comparative perspective. In the study of Van Tubergen et al. (2004), the ratio of income inequalities in destination and origin countries was positively associated with immigrants’ integration in the labor market, suggesting that high-inequality countries attract especially high-ability migrants. Also, academic achievement of children of immigrants to traditional migration societies with strict immigration policies (such as the USA and Canada) is higher than of immigrants to more recent host societies with more relaxed policies regarding the human capital of migrants (Levels et al. 2008). That same study also showed that (parental) educational disadvantage of first-generation immigrants relative to natives of the destination country harms academic achievement of school-aged immigrants’ children. Thus, poorly educated immigrant communities are harmful to children’s educational performance, independent of the socioeconomic status of the family.

Another indirect approach to assessing selectivity is to compare student test scores of migrant groups with the test scores of non-migrants in the origin country (Dronkers and De Heus 2010). For many immigrant groups, it appeared that their children performed worse than the majority children in the origin country, a finding that persisted after controlling for social class differences. However, it is uncertain whether such differences result from selective migration, or from difficulties in completing achievement tests in a non-native language (Heath and Kilpi-Jakonen 2012). It is somewhat worrying that most immigrant groups are, according to this approach, negatively selected, while educational selectivity is typically positive in other approaches.

We expect that more positively selected immigrant communities have lower disadvantages in education (relative to the majority populations in the destination countries) in comparison with more negatively selected immigrant communities (*hypothesis 1*).

### Destination Country Institutions

While comparative studies have shown some indirect evidence for the relevance of selective migration on integration, to the best of our knowledge, no earlier comparative study has directly measured the implications of selective migration for children’s educational attainment. A comparative approach is highly relevant, because destination countries vary significantly in how immigrants are incorporated into host societies. It allows us to investigate whether selectivity has different effects in migrant-friendly societies compared to more restrictive host societies.

Various scholars have drawn attention to the way in which host country policies and practices may affect the ‘warmth of the welcome’ afforded migrants and their prospects for successful integration, particularly focussing on economic integration (Reitz 1998; Portes and Zhou 1993; Heath and Cheung 2007). Such policies include anti-discrimination legislation, access to employment, citizenship and long-term residence, and practical assistance for recently arrived migrants and can be thought of as policies which tend to favor the inclusion rather than exclusion of migrant communities. A special form of integration policies are multicultural policies, an overlapping but conceptually distinct set of policies which facilitate recognition of ethno-religious groups and their distinctive cultures (Koopmans et al. 2005; Koopmans 2010; Banting and Kymlicka 2006; Wright and Bloemraad 2012; Bloemraad and Wright 2014). One perspective stresses that multicultural policies would improve the integration of migrants into host societies (Banting and Kymlicka 2006; Wright and Bloemraad 2012). However, others have argued that multicultural policies may in fact stigmatize migrant groups and hence deteriorate their integration in society (Duyvendak and Scholten 2012; Koopmans et al. 2005). Maybe both effects are in operation, leading to a nonexistent association between multicultural policies and the level of disadvantage. It is clear that comparative studies disagree on the relevance of host country institutions such as integration policies for educational integration (Rothon et al. 2009; Hillmert 2013). To make progress in this discussion, it is relevant to study the relation between integration policies and the relevance of selectivity of migration.

It is likely that the extent to which selective migration is associated with educational disadvantages depends on migrant-friendly integration policies. Warmth-of-welcome policies may be particularly important for children of ‘negatively selected’ migrant communities, i.e., with lower levels of human capital than the non-migrating homestayers. These groups are often targeted by integration-friendly policies. Based on this reasoning, we can formulate the hypothesis that the association between educational selectivity of immigrant communities and children’s reduced educational disadvantage is weaker in societies with inclusive migrant integration policies (*hypothesis 2*). In those migrant-friendly societies, strongly positively selected immigrant communities are less helpful for children’s educational attainment than in destination countries more hostile to immigrants.

A second destination country institution relevant for ethnic educational inequality concerns the tracking of the educational system in the host societies (Cobb-Clark et al. 2012; Crul and Vermeulen 2003; Entorf and Lauk 2008; Griga and Hadjar 2014). Education in the destination country is one of the key institutions that determine how context helps or hinders migrants’ children’s integration (Cobb-Clark et al. 2012; Crul and Schneider 2009, 2010). Educational systems vary strongly in the timing of selection into different school types. In Germany, students are selected into different school types, often in different school organizations, at the age of 10. In the Netherlands, this happens at the age of 12. These are early selecting countries. On the other hand, in countries like Sweden and Canada, selection into different school types occurs much later.^1^

Early tracking magnifies inequalities in educational achievement and school type enrollment because second-generation pupils are given ‘little time to pull themselves out of their disadvantaged starting position’ (Crul and Vermeulen 2003: 979).

Tracking is likely to have different effects for different groups of migrants, depending on their human capital selectivity. Through mechanisms of peer effects, resource allocation, and teacher quality, early tracking is particularly harmful for the educational opportunities of students placed in the less demanding tracks. Students in the higher tracks may benefit from early selection. How does tracking affect students of different levels of selection on the basis of human capital? A first line of reasoning is that tracking is particularly harmful to disadvantaged groups, so negatively selected immigrant communities (*hypothesis 3a*). More positively selected migrant groups may, according to this perspective, be less harmed by early tracking; also in early tracking systems, they may find their way to higher levels of achievement and attainment. Support for this reasoning may come from the consistently high levels of performance of Asian-origin students across many societies (Heath and Brinbaum 2014).

However, an alternative perspective is that children with a lot of learning potential and aspirations (i.e., coming from highly motivated and positively selectively migrated communities) will be particularly harmed by early tracking. They are the ones for whom early tracking limits the time to demonstrate their learning potential. For unselective, more disadvantaged students, tracking may be less influential on their educational career, as they would have had a lower performance independent of whether they are tracked early or not. Tracking would then effectively constrain people with high aspirations but initial low achievement, whereas non-selective systems allow them to continue. An alternative hypothesis therefore is that tracking reduces the positive influence of positive selection, as it will prevent positively selected groups from following their high aspirations (Waters et al. 2013) (*hypothesis 3b*).

Although we do not explicitly formulate hypotheses on differences in the statistical relationships across the educational career, it is possible to speculate on this issue. From one perspective, it can be expected that various indicators of disadvantage have stronger effects early in the school career. Many studies have reported reduced effects across the career, for two reasons: because there is an increased homogeneity of students after rounds of selection in the education trajectory and because children are more independent of their parents’ structural position at higher ages (Breen and Jonsson 2005). If the effects (which are our dependent variables) are smaller, this translates into lower variances in our dependent variable, potentially leading to lower statistical relationships. However, given that we study unconditional models, meaning that there is no increased homogeneity on unobservables across the outcomes, it could also be that institutions affecting test results at the beginning of secondary education have persistent effects across the career.

## Research Design

### Data

Our analysis concentrates on gaps in educational outcomes between the second generation and majority groups. The second generation is defined as the group that is born in the destination country, but with at least one parent born abroad. If one parent was born abroad, the origin country of that parent was used to identify ethnic group. This means that we define ethnic background based on country of origin of the parent(s), which, we realize, can be a simplification if multiple ethnic groups can originate from one origin country.

We focus on ten host countries in Europe and North America: Belgium, Canada, England, Finland, France, Germany, the Netherlands, Sweden, Switzerland, and the USA. We brought together expertise from these countries in a European research project funded under the EQUALSOC Network of Excellence (a Sixth Framework Programme funded by the European Commission) and reported in Heath and Brinbaum (2014). We included countries where a sizeable second generation has already gone through the complete school system. The ten countries have all become increasingly diverse in the past decades, and the set of countries includes important variations with regard to selective migration, socioeconomic origin and destination country institutions. For reasons of data availability, for Switzerland, we only included the first outcome (test scores). We maintained Switzerland because our team included expertise on Switzerland, and because including it is relevant to study the impact of school tracking, one of our contextual variables.

We selected nationally representative datasets to study ethnic educational inequalities at various stages in the educational career and focused on what the national experts considered the best available datasets to study ethnic educational inequality in their country. We focus on three outcomes in the secondary school phase: (1) test scores at the first stage of secondary education (roughly at the age of 13–16), (2) enrollment in vocational or general/academic tracks in upper secondary education, and (3) the completion of upper secondary education. Based on available data and national expertise to analyze the first step in the analysis (see below), we were able to collect data on ten host countries, including traditional receiving societies (e.g., Canada, USA), countries with immigration from former colonies (e.g., France, England), countries with a major ‘guest worker’ source of immigration (e.g., Germany, the Netherlands), and countries with a large refugee population (e.g., Sweden). Moreover, these countries differ in substantial ways with regard to their institutions that may be influential on the role of selective migration. The data we brought together include cohort studies of educational or birth cohorts, register data, and cross-sectional data. This approach has the advantage that the best datasets are chosen that have been used for national studies of ethnic and social inequalities in education, many of which are longitudinal and collected by national statistical agencies. A disadvantage is that the data were initially not collected for comparative purposes. Datasets varied in some notable respects between countries. For Sweden, Finland, and Belgium, population-level data were obtained from official registers, while for other countries, cross-sectional data were combined with educational cohort studies. Nevertheless, all these datasets were nationally representative. Given that the data came from various sources (including national registers), there was no possibility to bring the individual-level data together.

Not all educational outcomes are available for all ten host countries. See "Appendix 1" for an overview of the dependent variables that have been studied per country and of the ethnic groups that have been compared to majority populations. "Appendix 2" lists all the datasets that have been used.

### Estimation Strategy

We use a two-step procedure to assess the relationships between the contextual variables and ethnic educational inequality. Ethnic educational inequality is measured by the net regression coefficient of ethnic group, for each ethnic group and each destination country separately, on the outcome under study. The first step consists of country-by-country regressions on the three outcomes, controlling for family situation (single parenthood), social background (parents’ education and social class), and gender. These models are unconditional models; for instance, the chance to obtain a full secondary-level qualification is estimated for the whole sample, not just for people that have successfully completed lower secondary education.^2^

Of these regression models, the coefficients displaying the difference in the outcome of a particular ethnic group with children from the majority population are saved, as well as the standard errors of these coefficients. With regard to test results, these coefficients are taken from ordinary least squares regression models. For the other two outcomes, which are dichotomous, we have taken the probit coefficients. Negative net coefficients would thus indicate a disadvantage of that particular ethnic group for that particular educational outcome compared to similar children of majority populations, and positive coefficients represent net advantages.

This paper reports about the second step of the regression analysis, which relates the strength of the (dis)advantage of second-generation students to contextual variables such as integration policies that are described below. The dependent variables are, thus, the *coefficients* of ethnic background predicting the educational outcome, which indicate the level of (dis)advantage of the particular ethnic group relative to the majority population, controlled for socioeconomic background (occupational group and both parents’ education). These models are known as slopes as outcomes models or two-step multilevel models (Achen 2005; Bryan and Jenkins 2016; Gebel and Giesecke 2011, 2016).

These coefficients measure the ethnic gap of ethnic group coming from origin country *j* in destination country *k* (relative to the majority population), which we call *δ*_*jk*_. We estimate the size of the gap as a function of variables at the level of destination country *k* and of the combination of origin and destination countries *{j,k}*, and of an interaction term between these two different types of variables. Equation (1) describes the second-step multilevel regression model, with ethnic groups nested in host societies (estimated separately for each educational outcome):

1 $$\delta_{jk} = \alpha + \beta X_{k} + \gamma Z_{jk} + \tau X_{k} Z_{jk} + \zeta_{k} + \varepsilon_{jk}$$

Since our dependent variable *δ*_*jk*_ is an *estimated* coefficient rather than the true population-level parameter, we take into account the degree of uncertainty of the coefficients by weighting. We apply the weighting scheme proposed by Borjas and Sueyoshi (1994), which takes into account the two error components that exist in two-stage models (see Huber et al. 2005). The first component is the variance of the coefficients estimated in the first-stage regressions. This variance is easily derived on the basis of the standard errors of the first-stage coefficients, in such a way that more precise estimates get greater weights than more imprecise estimates. The second component is the residual variance from the second-stage regressions before adding weights—that is, the residuals of this model net of the variance resulting from the first-stage coefficients. This second component is necessary because the macro-level variables do not explain the whole variance between countries.

Due to the relatively low number of cases (i.e., ethnic communities), we are careful not to include too many explanatory variables at once. Therefore, each analysis is built up in steps. The main predictor variables are the selective migration index (see below), which is subsequently interacted with two destination country variables: an indicator for multicultural policies, and an indicator of early tracking in the educational system. We control for language similarity of the origin and host societies, as it is shown to be related to both the selectivity of migration (Brücker and Defoort 2009) and educational attainment (Heath et al. 2008).

**Fig. 1:**
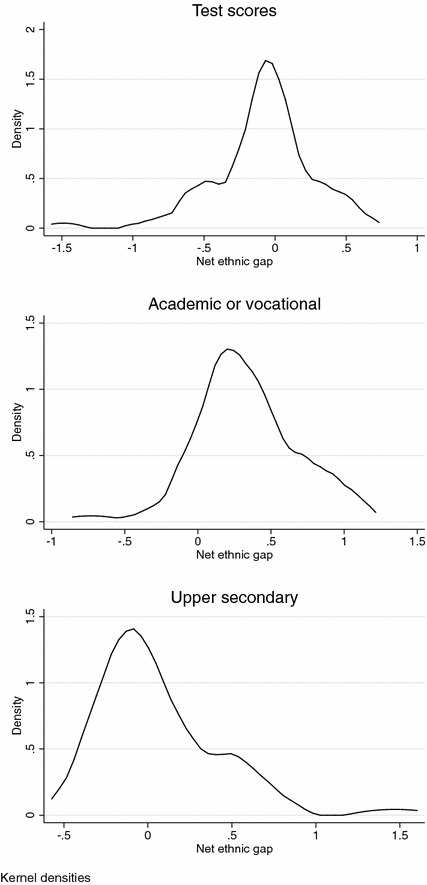
Net effects of ethnic background on educational outcomes (*δ*_*jk*_)

The dataset has ethnic group by destination country as its unit of analysis. With 32 ethnic groups in ten countries (including an ‘other’ and ‘mixed’ category in each of the countries), the total N lies between 57 and 80 (depending on the specific educational outcome). Figure 1 shows the kernel distributions of the net effects on the three educational outcomes. It can be seen that both negative and positive net effects are found in the data. In "Appendix 4", we show models on the subset of the countries that are available for all three outcome variables. In the conclusion and discussion, we reflect on the robustness of our findings in light of these replications on smaller datasets.

### Selectivity of Migration

We follow the approach of Feliciano (2005a) in measuring selectivity of migration. A selectivity index is constructed to indicate how the educational level of migrants compares to their origin countries’ populations from the same birth cohort (in order to take account of the fact that educational levels have been rising rapidly in many of the ‘origin’ countries just as it has in the Western destination countries). Data on (first-generation) fathers’ completed education are readily available in the datasets used in the first-step analysis.^3^ The host country data are taken from nationally representative data among children, so first-generation migrants without children are not represented. For the non-migrant population of origin countries, data were gathered from several (cross-) national surveys. Like Feliciano, we use the net difference index (NDI) introduced by Lieberson (1976, 1980) to calculate a measure of selectivity. This index enables us to compare the entire frequency distribution of completed educational level by migrant fathers to that of non-migrant males of a comparable age-group in the origin country.^4^ Its logic is to sum up the percentages of migrants in the destination country that completed a similar, lower, and higher educational level in comparison with their non-migrant counterparts in the origin country. The sum score represents how often the educational level of a migrant will exceed that of a non-migrant, or the other way around. The more positive the sum score, the more the educational level of a migrant group exceeds that of a non-migrant as opposed to the other way around. A negative sum, in turn, indicates that migrants more often have a lower educational level than non-migrants. The selectivity index ranges from − 1 (all migrants are less educated than non-migrants) to + 1 (all migrants are more educated than non-migrants). A score of 0 means that the educational distributions of migrants and non-migrants are equal.

Selectivity cannot be calculated for the ‘mixed’ and ‘other’ ethnic groups since these categories have no specific origin country. For the ‘other’ category, the mean selectivity index of the destination country is imputed. For the ‘mixed’ category, we impute a selectivity of 0, equating this category to the majority group. It should be noted that this measure only assesses selectivity with regard to educational attainment. Other forms of selectivity, for instance on the basis of intelligence, motivation, or economic resources, are not explicitly measured, while these could be influential on the level of disadvantage in immigrant communities.

**Fig. 2:**
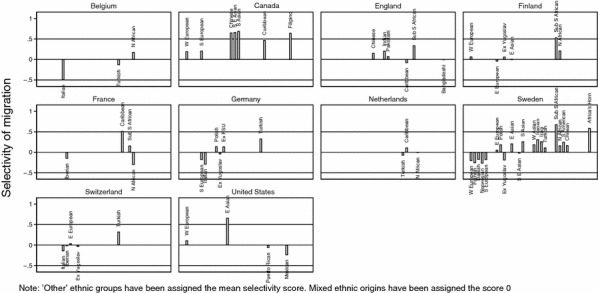
Selectivity of migration by ethnic group and host country

Figure 2 shows the selectivity index calculated for each origin group in each of the destination countries. The figure shows that selectivity varies strongly between ethnic groups within countries, and within ethnic groups between countries. For instance, the Turkish first-generation migrants living in Germany are more positively selected than Turkish migrants in the Netherlands or Belgium (while Zuccotti et al. 2017 showed overall positive selection of Turkish migrants in six European destination countries). Moreover, in line with Canadian policy, it is evident that all migrant groups that were examined are positively selected (i.e., more highly educated than the homestayers in the origin countries). All other countries have both positively selected and negatively selected migrant groups.

The control variable language similarity between origin and destination countries is measured as a dummy variable where a score of 1 signifies similarity of languages. Ethnic groups subsumed under the ‘other’ category are given a 0. The ‘mixed’ category is scored 1.

### Destination Country Institutions: Integration Policies and Early Tracking

At the level of the destination country, one central variable measures the extent to which the country has policies aimed at promoting migrant integration. We use the Migrant Integration Policy Index of 2010 (MIPEX III, MPG 2011), which can be thought of as an overall index of the inclusiveness of host country institutions; the MIPEX index aims to ‘create a rich, multi-dimensional picture of migrants’ opportunities to participate in society by assessing governments’ commitment to integration’ (MPG 2011: 6). On the MIPEX Web site, the aims of the index are more elaborately described as follows: ‘MIPEX measures policies that promote integration in all societies. Integration in both social and civic terms rests on the concept of equal opportunities for all. In socio-economic terms, migrants must have equal opportunities to lead just as dignified, independent and active lives as the rest of the population. In civic terms, all residents can commit themselves to mutual rights and responsibilities on the basis of equality. When migrants feel secure, confident and welcome, they are able to invest in their new country of residence and make valued contributions to society. Over time, migrants can take up more opportunities to participate, more rights, more responsibilities and, if they wish, full national citizenship.’^5^ Using expert surveys and policy assessments, seven dimensions of integration policies are quantified: labor market mobility, family reunion, education, political participation, long-term residence, access to nationality, anti-discrimination. Countries are scored according to the extent to which migrants have equal opportunities as the rest of the population. It can be thought of as a measure of the overall ‘warmth of the welcome.’ It should be noted that some elements of MIPEX may be less relevant for migrants within the European Union, as they are entitled to free movement and residence. Nevertheless, other elements such as entitlement to full national citizenship and rights to participate can also vary with regard to European migrants and their families, depending on the host country.

It is important to note that the explicit focus is on policies, not on factual integration levels. We have included the total MIPEX score across all dimensions, rather than focus on education policies alone, because the reduction in educational disadvantages is likely affected by broader sets of policies to integration of families, including their labor market, political and civic integration.^6^ The MIPEX index correlates strongly with multiculturalism indicators (Koopmans et al. 2012).^7^

The *tracking* of the educational system is measured by a standardized scale based on age of first selection, the length of the differentiated curriculum, and the number of school types available for 15-year old students (taken from OECD reports and Brunello and Checchi 2007, see Bol and Van de Werfhorst 2013; Bol et al. 2014). Factor scores of an underlying factor were calculated and standardized with a mean of 0 and a standard deviation of 1, taken over the maximum number of countries available in the source data. Summarizing different indicators of tracking into one index is important, as together they indicate the timing (i.e., at which age), duration (which proportion of the compulsory years of education), and form of tracking (i.e., in how many tracks students are separated). Regression diagnostics of the association between tracking and the slope of family background on student achievement are more supportive of the composite index than of the separate underlying indicators [Authors]. It should be noted that the tracking index classifies educational systems particularly with regard to differentiation in the first stage of secondary education, thus before the outcome that we study of whether children enroll the academic or vocational track in upper secondary education.

Table 1 shows the scores on all the contextual variables, averaged across ethnic groups within host countries. Regarding selectivity of migration, we see that the average selectivity is larger than zero in all ten destination countries, indicating positive selection on educational attainment of the first-generation migrants relative to the homestayers. As one would expect based on selective immigration policies, the highest value is found in Canada, where the average selectivity is 0.406. A number of countries have an intermediate selectivity index of around 0.10, including England, Finland, Sweden, and the USA. Then, countries that adopted many ‘guest workers’ in the 1960s and 1970s (Belgium, Germany, the Netherlands) have lower selectivity scores.

**Table 1:** Descriptive statistics explanatory variables per country (based on dataset for test scores)

Country	Selective migration index	Same language	Migrant Integration Policy Index (/10)	Tracking index
Belgium	0.043	0.250	6.7	1.041
Canada	0.406	0.333	7.2	− 1.315
England	0.101	0.625	5.7	− 1.078
Finland	0.120	0.125	6.9	− 0.930
France	0.058	0.667	5.1	− 0.477
Germany	0.026	0.125	5.7	1.789
The Netherlands	0.038	0.250	6.8	0.971
Sweden	0.101	0.048	8.3	− 1.058
Switzerland	0.040	0.143	4.3	− 0.024
USA	0.093	0.400	6.2	− 1.315

These statistics are unweighted for group sizes

Language similarity is highest in France and England, countries where many migrants from former colonies moved to. The migrant integration policy index is highest for Sweden and also comparatively high in Finland, the Netherlands, and Belgium. In France, Germany, and Switzerland, migration integration policies are less inclusive. Educational tracking is highest in the German educational system, where students are typically selected around the age of 10 for three separate school types. Also Belgium and the Netherlands score high on tracking, with selection happening around the age of 12, for separate school careers for multiple years. Less tracking is found in the comprehensive schooling systems in Scandinavia, England, Canada, and the USA.

## Results

The outcomes of our two-step multilevel models with additional clustering of migrant groups within countries are shown in Table 2. The table lists five models for each of the three educational outcomes. First, we start with a model with only selective migration and language similarity. Then, the migration policy index is added (model 2) plus interaction terms with selectivity of migration (model 3). Then, in models 4–5, the migration policy index is replaced with tracking; first the main effect of the variable at the destination country level is inserted, after which interaction term is added.

**Table 2:** Multilevel regression coefficients of selectivity of migration, language similarity, and destination country institutions on the level of ethnic inequality in three educational outcomes^a^

	(1)	(2)	(3)	(4)	(5)
*Net ethnic gap in student test scores*					
Selective migration index	0.311***	0.174**	0.297	0.136∼	− 0.042
	(3.39)	(2.67)	(0.58)	(1.82)	(− 0.59)
Same language in origin and destination countries	0.159	0.258*	0.257*	0.113	0.118
	(1.12)	(2.23)	(2.20)	(0.91)	(0.94)
Migrant integration policy (MIPEX/10)		0.154***	0.155***		
		(3.34)	(3.38)		
Selective migration index * (MIPEX/10)			− 0.018		
			(− 0.27)		
Tracking index				− 0.187**	− 0.180**
				(− 2.87)	(− 2.85)
Selective migration index * Tracking index					− 0.214*
					(− 2.01)
Constant	− 0.173	− 1.186**	− 1.193***	− 0.230∼	− 0.231∼
	(− 1.10)	(− 3.25)	(− 3.32)	(− 1.76)	(− 1.80)
Variance between countries	3.08e−23	5.00e−23***	1.25e−24	7.61e−17***	8.25e−23
	(− 0.38)	(− 7.28)	(− 0.29)	(− 5.96)	(− 0.26)
Variance between ethnic groups within countries	0.146***	0.101***	0.101***	0.114***	0.112***
	(− 3.91)	(− 8.68)	(− 5.55)	(− 4.90)	(− 4.04)
Observations	80	80	80	80	80
*Net ethnic gap in academic versus vocational track*					
Selective migration index	0.727***	0.727***	− 1.071∼	0.727***	0.610***
	(7.12)	(7.09)	(− 1.75)	(7.09)	(5.58)
Same language in origin and destination countries	− 0.055	− 0.054	− 0.044	− 0.055	− 0.050
	(− 0.69)	(− 0.69)	(− 0.53)	(− 0.69)	(− 0.63)
Migrant integration policy (MIPEX/10)		0.083*	0.070∼		
		(2.39)	(1.87)		
Selective migration index * (MIPEX/10)			0.230**		
			(3.08)		
Tracking index				− 0.095***	− 0.088**
				(− 3.55)	(− 3.22)
Selective migration index * Tracking index					− 0.150**
					(− 2.71)
Constant	0.219**	− 0.317	− 0.219	0.222***	0.223***
	(3.00)	(− 1.38)	(− 0.89)	(4.68)	(4.42)
Variance between countries	0.017***	0.010***	0.011***	0.006***	0.006***
	(− 6.95)	(− 18.95)	(− 17.69)	(− 8.14)	(− 8.22)
Variance between ethnic groups within countries	0.047***	0.047***	0.044***	0.047***	0.047***
	(− 22.81)	(− 22.72)	(− 20.12)	(− 22.72)	(− 22.73)
Observations	60	60	60	60	60
*Net ethnic gap in completion of upper secondary education*					
Selective migration index	0.184	0.184	1.187	0.184	− 0.457
	(0.97)	(0.97)	(1.13)	(0.97)	(− 0.71)
Same language in origin and destination countries	− 0.094∼	− 0.094∼	− 0.100*	− 0.094∼	− 0.073
	(− 1.89)	(− 1.89)	(− 2.00)	(− 1.89)	(− 1.35)
Migrant integration policy (MIPEX/10)		− 0.068	− 0.058		
		(− 1.22)	(− 1.09)		
Selective migration index * (MIPEX/10)			− 0.136		
			(− 1.05)		
Tracking index				− 0.177***	− 0.162**
				(− 3.64)	(− 3.20)
Selective migration index * Tracking index					− 0.589
					(− 0.97)
Constant	0.080	0.530∼	0.459	− 0.002	− 0.004
	(0.76)	(1.68)	(1.56)	(− 0.04)	(− 0.08)
Variance between countries	0.056***	0.051***	0.050***	0.027***	0.023***
	(− 6.87)	(− 5.06)	(− 5.11)	(− 5.96)	(− 6.35)
Variance between ethnic groups within countries	0.053***	0.053***	0.051***	0.053***	0.051***
	(− 7.45)	(− 7.46)	(− 7.82)	(− 7.46)	(− 7.90)
Observations	57	57	57	57	57

*t* statistics in parentheses

∼*p *< 0.10; *p < 0.05; **p < 0.01; ***p < 0.001

^a^The unit of observation is migrant group within destination country

The first outcome is ethnic educational inequality in student test scores. Table 2 shows that selectivity of migration is positively associated with the coefficient of ethnic background relative to the majority population, in line with hypothesis 1. Given the value of the constant, the average disadvantage of unselective groups coming from countries with a different language is − 0.173 standard deviations in test scores. If selectivity increases, the gap is reduced and could turn even into an advantage (given the maximum score of selectivity of, roughly, 0.7). Language similarity is also associated with reduced disadvantages, although the coefficient is not statistically significant.

In model 2 where the MIPEX migrant integration policy index is added, the coefficient of language similarity is increased and turns into significance, while the positive coefficient for selectivity of migration gets weaker, but also stays significant. The MIPEX is positively associated with the ethnicity coefficient, meaning that disadvantage in student test scores is lower in countries with more favorable migrant integration policies.

Model 3 adds the interaction term between MIPEX and selectivity of migration, but that turned out to be irrelevant and statistically insignificant (falsifying hypothesis 2). So, while migrant integration policies are associated with lower disadvantages among migrants relative to majority populations, this relationship is highly similar across ethnic groups of different levels of selectivity.

Model 4 replaces the MIPEX index with the tracking index of the educational system. The overall effect of tracking is negative; in more strongly tracked educational systems, the average level of disadvantage of ethnic groups is larger than in countries with comprehensive schooling systems. Model 5 shows that the negative effect of tracking gets even more negative for more strongly selected migrant groups—a finding in line with hypothesis 3b. The interaction term is pretty strong, but the interaction effect is not replicated if we only focus on countries for which we study all three dependent variables (Table 6 of Appendix). It is replicated, however, if only European countries are analyzed (Table 7 of Appendix).

The second panel of Table 2 shows results of an analysis of ethnic differences in the choice for general/academic forms of upper secondary education, relative to vocational education. As already seen in Fig. 2, the overall level of disadvantage is smaller than with test scores, and in fact many ethnic groups have a net advantage over children of the majority population with similar socioeconomic backgrounds. The intercept has a positive value, indicating an average advantage for children from non-selective communities from non-native-speaking origin countries (i.e., overrepresentation of second-generation migrants in the general/academic programs). Model 1 shows that selective migration is positively associated with the size of the regression coefficient of ethnic background. More selectively migrated communities have higher likelihoods to be enrolled in the academic tracks in upper secondary education. This is in line with hypothesis 1. The MIPEX index is positively associated with the ethnicity slope, indicating higher levels of ethnic advantage (or lower disadvantage) in societies with more migrant-friendly policies.

Model 3 shows that selective migration is even more positively associated with minorities’ opportunities in education in countries with favorable migrant integration policies. In other words, migration integration policies are particularly helpful for communities that are strongly positively selected, which goes against hypothesis 2. One interpretation of this finding may be that favorable integration policies are particularly helpful for positively selected groups with high aspirations. It is easier for them to take advantage of the opportunities available in favorable integration regimes.

Model 4 shows that early tracking is associated with larger disadvantages (or smaller advantages) of ethnic minorities relative to majority populations with regard to upper secondary academic enrollment. So, in countries where tracking happens earlier and more rigidly at the first stage of secondary education, there is a weaker overrepresentation of second-generation immigrants in the academic schools at the upper secondary level. This finding corresponds to the multination comparison of student achievements at age 15 (Cobb-Clark et al. 2012), but is now further supported using post-harmonized educational cohort data for an outcome at the upper secondary level.

Model 5 shows that tracking is particularly harmful to students coming from more strongly selected migrant communities. Like with student test scores, this is in support of hypothesis 3b, which argues that tracking is particularly harmful to ambitious and motivated migrant communities.

The third panel of Table 2 shows results on the completion of full upper secondary education (in any track). Overall, the results are less strong than in the previous analyses, possibly because different datasets have been used, and fewer countries could be included (see "Appendix 2"). For instance, for the Netherlands, we now have to rely on the educational attainment of young adults in a cross-sectional survey, while for previous analysis, we used prospective educational cohort data. Also for Britain, other data are used.

There is no significant association between selective migration and secondary degree completion, although the coefficient is positive as in previous analyses. Model 2 shows that migrant integration policies are not associated with secondary degree completion. So, while the previous panel showed a positive association with the enrollment into academic forms of secondary education, we do not see that this translates into lower inequalities in terms of the completion of secondary education. The coefficient for the migrant integration policy index is unrelated to the selectivity of migrant communities (model 3).^8^

Model 4 shows that early tracking magnifies the ethnic educational inequalities in secondary degree completion. So, like with test scores and the choice for the academic track, tracking is associated with larger ethnic gaps. Model 5 shows that the interaction term between tracking and selectivity is negative, as with the previous two dependent variables, but in this case, the association is not statistically significant.

## Conclusion and Discussion

We studied ethnic educational inequalities among second-generation migrants in ten destination countries, concerning three crucial outcomes in educational careers: test results, choosing the academic or vocational route in upper secondary education, and the completion of upper secondary education. With our career perspective on ethnic inequalities in education, holding constant for socioeconomic differences between groups, we examine whether ‘context matters’ for the integration and assimilation of migrant children in the secondary educational system.

Building on the perspective that ethnic communities are defined based on the combination of the country of origin and the country of destination of migrants (Van Tubergen et al. 2004), our focus was on the question whether selective migration of a migrant group relative to the non-migrating ‘home stayers’ is related to the level of disadvantage in education. We found support for the baseline hypothesis that more positively selected migrant communities have lower levels of disadvantage (or a higher level of advantage) relative to the majority population. This effect was only found to be statistically significant for the standardized tests taken at the first stage of secondary education and for the choice of academic versus vocational education. For secondary school completion, the effect of selectivity of migration was more modest and had higher levels of statistical uncertainty. Possibly, differences across educational outcomes imply that the ethnic community seems more important for educational outcomes in the early school career than for outcomes later in the career.

We also examined whether selective migration was differentially associated with ethnic disadvantage in education depending on host country institutions, in particular migrant-friendly policies and the early tracking of the system. First of all, it appeared that ‘context matters’ in that the minority–majority education gaps were associated with these institutional characteristics. Gaps were less negative for minority students (or more positive, as the net ethnic gaps are often favorable for the second generation when socioeconomic differences are taken into account) in societies with migrant-friendly policies—again with the exception of the likelihood to complete upper secondary education. Gaps were more negative (or less positive) in societies with strongly tracked educational systems, and consistent so for all studied outcomes. However, the evidence that these institutional environments had differential associations with ethnic inequalities depending on the selectivity of migration is less clear. If anything, we see that tracking in education is particularly harmful to positively selected immigrant communities, but the effect is not robust to specific country selections. In terms of policy implications, the ‘warmth of the welcome’ and the tracking of the system seem to matter, but there is, based on our findings, little reason to think that these institutional characteristics matter differently depending on the migrant group’s selectivity.

Our focus on tracking does not mean that this is the only form of school segregation that may impact children of immigrants disproportionately. Other forms of segregation may be manifested through residential segregation, or through private schools as apparent in the USA and England. Nevertheless, the private sector only includes around 10 percent of primary and secondary school children in the USA (McFarland et al. 2017) and 9 percent in England (Department for Education 2016), while academic/general tracks typically have an intake of around 40–50% of students. Nevertheless, it is worthwhile to further study the impact of private education on students of different migration backgrounds.

This study’s findings are important for at least three fields of investigation. First of all, while there are many studies that have aimed to explain the extent to which migrant communities are positively selected based on human capital, few studies have examined the implications for the level of ethnic educational inequality, let alone inequalities at various stages of the educational career. Showing the implications of selectivity of migration further emphasizes the relevance of studying the migration process itself. As a follow-up to our study, a multi-origin-multi-destination design can also examine the educational distribution in the home country in a different way. As Feliciano and Lanuza (2017) show, the educational attainment of immigrants may also be seen as a positional good, where a medium-level qualification may in fact represent a comparatively high achievement level, depending on the origin country.

Second, our study has contributed to the understanding of ethnic educational inequalities, by emphasizing the relevance of the context of reception. The observed level of selectivity of migration is a consequence of two joint processes: (1) selectivity of who migrates and (2) the choice of country to migrate to. This makes that we have to be careful to think of the associations of selective migration as causal. Nevertheless, given that the destination of migrants is at least partially a non-rational random process, it is worthwhile to study context in relation to selectivity of migration. Moreover, if an optimal sorting process would have occurred, no differential effects would have been found as each community would have ended up in the best host country. Nevertheless, a possible weakness of our design is that our findings may result from ethnic communities being conducive to educational attainment of children on top of individual socioeconomic background, while selective migration has no causal effect on a counterfactual interpretation of what would have happened had migration not taken place.

A third field of enquiry to which our study speaks is concerned with the impact of the educational institutional structure on various inequalities. While most of the literature on educational systems is concerned with social, rather than ethnic, inequalities (Brunello and Checchi 2007; Van de Werfhorst and Mijs 2010), some studies have pointed to larger ethnic inequalities in early tracked educational systems (Cobb-Clark et al. 2012, Crul and Vermeulen 2003). Our data found evidence for this as well, studying multiple educational outcomes. For all three educational outcomes, ethnic minorities were further disadvantaged in societies with early tracking systems (cf. Griga and Hadjar 2014 for tertiary education). Moreover, early tracking was particularly harmful to strongly positively selected ethnic communities. Tracking thus seems to harm the educational potential specifically of highly motivated and ambitious ethnic groups.

In summary, selectivity of migration appeared a relevant correlate of the educational performance differentials between the second generation and majority populations. Although our data do not contain measurements of aspirations and motivations, these offer relevant interpretations of this central finding. Positively selected migrant groups do better in education than migrant groups that have been less positively selected. Migrants are, according to the immigrant optimism thesis of Kao and Tienda (1995), anxious and motivated to integrate in their new country, and their optimism stimulates their children’s schooling outcomes. This particularly affects ethnic communities that form a positive selection of their origin country’s population in terms of human capital.

Finally, it is worth mentioning some weaknesses of our study. With our approach to select the best possible data per country, each with sizeable immigrant communities, we had to harmonize the data after data collection, which always comes at a cost with regard to comparability. One disbalance is that the countries differed in the number of immigrant groups that were included. It is possible that our results are partly driven by the countries with many immigrant groups, most notably Sweden. Another disadvantage of post-harmonization is that some datasets are longitudinal and others are not. Also, our two-step design required many ‘cases,’ necessitating us to combine all countries and all origin countries. Robustness checks with fewer countries sometimes refuted the overall findings, but particularly with regard to the interaction of destination country institutions by selectivity of migration. Finally, our approach to measure selective migration conflates selection processes in the decision to migrate and the destination where to move. Our study can be seen as complementary to studies that more specifically address these joint decisions in the migration process (Grogger and Hanson 2011; Guveli et al. 2016).

## Appendix 1

See Table 3.

**Table 3:** Ethnic groups by country

	Number of ethnic groups	Ethnic groups studied per outcome variable^a^
Belgium	5	Outcomes 1, 2, 3 Italian, Turkish, North African, other, mixed
Canada	9	Outcomes 1 and 3 Western Europe, Southern Europe, Chinese, South East Asian, South Asian, Caribbean, Filipino, other, mixed
England	8	Outcomes 1, 2, 3 Chinese, Indian, Pakistani, Caribbean, Sub-Saharan African, Bangladesh, other, mixed
Finland	8	Outcomes 1, 2 Western Europe, Eastern Europe, Ex-Yugoslavian, East Asian, sub-Saharan African, North African, other, mixed
France	6	Outcomes 1, 2, 3 Iberian, Caribbean, Sub-Saharan African, North African, other, mixed
Germany	8	Outcomes 1, 3 Southern European, Italy, Polish, Ex-Yugoslavian, Former Soviet Union, Turkish, other, mixed
The Netherlands	4	Outcomes 1, 3 Turkish, Caribbean, North African, other Outcome 3 Turkish, Caribbean, North African
Sweden	21	Outcomes 1, 2, 3 Western European, Finnish, Danish, Norwegian, Southern European, Eastern European, Polish, Ex-Yugoslavian, East Asian, South East Asian, South Asian, West Asian, Iranian, Iraqi, Turkish, Sub-Saharan African, North African, South American, Chilean, Africa’s Horn, mixed
Switzerland	7	Outcome 1 Italian, Iberian, Eastern European, Ex-Yugoslavian, Turkish, other, mixed
USA	5	Outcomes 1, 3 Western European, East Asian, Puerto Rican, Mexican, other, mixed

^a^Outcome 1: Student test scores, Outcome 2: Vocational or academic track within upper secondary education, Outcome 3: Completion of upper secondary education

## Appendix 2

See Table 4.

**Table 4:** Microdata that have been used in the first step of the analysis

	Outcome 1: Test scores	Outcome 2: Academic versus vocational within upper secondary education	Outcome 3: Completion of upper secondary education
Belgium	PISA 2003	Matched observations from Belgian census of 1991 (parental education, social class, ethnicity) and 2001 (educational outcomes)	
Britain	Longitudinal Study (linked census and vital event data of 1% of population of England and Wales),1991–2001	Youth Cohort Study cohort 10 sweep1 (2000)	Longitudinal Study 1991–2001
Canada	Youth in Transition Survey 2000	N/A	Youth in Transition Survey 2000
France	Panel 95 (Secondary school cohort data)		
Finland	Linked register data (Statistics Finland)		N/A
Germany	PISA-E 2000	Microcensus 2005 (West-Germany) (1% probability sample)	N/A
The Netherlands	VOCL 1999 (Secondary school cohort data)		Survey Integration of Minorities (SIM) 2006
Sweden	STAR database (Linked register data)		
Switzerland	PISA 2000	N/A	N/A
United States	Educational Longitudinal Study 2002	N/A	Educational Longitudinal Study 2002

*N/A* No data have been analyzed on this outcome for this country

## Appendix 3

See Table 5.

**Table 5:** Selective migration index per ethnic group and destination country

Host country	Ethnic group in data set	Specific origin country used	Data source on origin country	Selectivity index score
Canada	West European	UK	ESS 2002	0.194962
Finland	West European	UK	ESS 2002	0.066658
Sweden	West European	UK	ESS 2002	− 0.20931
USA	West European	UK	ESS 2002	0.11142
Sweden	Finnish	Finland	ESS 2002	− 0.26169
Sweden	Danish	Denmark	ISSP 2000	− 0.18047
Sweden	Norwegian	Norway	ESS 2002	− 0.27216
Canada	South European	Portugal	ESS 2002	0.209518
Germany	South European	Greece	ESS 2002	− 0.18499
Sweden	South European	Italy	ESS 2002/04/06 pooled	− 0.18499
Germany	Italian	Italy	ESS 2002-04-06 (pooled)	− 0.29638
Switzerland	Italian	Italy	ESS pooled	− 0.14623
Belgium	Italian	Italy	ESS pooled	− 0.49736
France	Iberian	Portugal	ESS 2002	− 0.15283
Switzerland	Iberian	Portugal	ESS 2002	− 0.02143
Finland	East European	Estonia	ISSP 2009	− 0.04595
Sweden	East European	Hungary	ISSP 2000 & ESS 2002 (pooled)	0.05488
Switzerland	East European	Albania	EVS 2008	0.037072
Germany	Polish	Poland	Pisa 2006	0.141243
Sweden	Polish	Poland	ESS 2007	0.18756
Finland	Ex-Yugoslavia	Croatia	ESS 2007	0.063322
Germany	Ex-Yugoslavia	Croatia	ESS 2007	− 0.04651
Sweden	Ex-Yugoslavia	Bosnia	BiH	− 0.19434
Switzerland	Ex-Yugoslavia	Croatia	ESS 2007	− 0.03873
Germany	Former Soviet Union	Russia	ISSP 2000	0.135678
Finland	East Asian	China	WVS 2–5	0.001543
Sweden	East Asian	China	WVS 2–5	0.210098
USA	East Asian	China	WVS 2–5	0.663011
Canada	Chinese	China	WVS 2–5	0.652187
England	Chinese	China	WVS 2–5	0.154286
Canada	South East Asian	Vietnam	WVS 4–5	0.663362
Sweden	South East Asian	Vietnam	WVS 4–5	− 0.0254
Canada	South Asian	India	SDSA	0.69034
Sweden	South Asian	India	SDSA	0.262061
England	Indian	India	SDSA	0.209051
England	Pakistani	Pakistan	SDSA	0.074064
Sweden	West Asian	Turkey	ESS 2007–2008	0.188775
Sweden	Iranian	Iran	WVS 4–5	0.312045
Sweden	Iraqi	Iraq	WVS 4–5	0.26471
Belgium	Turkish	Turkey	ESS 2007–08	− 0.13527
Germany	Turkish	Turkey	ESS 2007–08	0.329437
The Netherlands	Turkish	Turkey	ESS 2007–08	− 0.07772
Sweden	Turkish	Turkey	ESS 2007–08	0.1133
Switzerland	Turkish	Turkey	ESS 2007–08	0.323408
Canada	Caribbean	Jamaica	LAPOP 2010	0.472845
England	Caribbean	Jamaica	LAPOP 2010	− 0.08323
France	Caribbean	Dominican Republic	ISSP 2008	0.514558
The Netherlands	Caribbean	Suriname	LAPOP 2010	0.112146
USA	Puerto Rico	Puerto Rico	WVS 3–4	− 0.064
England	Sub-Saharan Africa	Nigeria	Afrobarometer 4	0.339071
Finland	Sub-Saharan Africa	Senegal	Afrobarometer 4	0.535145
France	Sub-Saharan Africa	Senegal	Afrobarometer 4	0.158402
Sweden	Sub-Saharan Africa	Senegal	Afrobarometer 4	0.678733
Canada	Sub-Saharan Africa	Nigeria	Afrobarometer 4	0.606676
Belgium	North African	Morocco	WVS 3–4 & Afrobarometer	0.179418
Finland	North African	Algeria	WVS 4	0.213563
France	North African	Algeria	WVS 4	− 0.30408
The Netherlands	North African	Morocco	WVS 3–4 & Afrobarometer	− 0.01006
Sweden	North African	Algeria	WVS 4	0.16425
Sweden	South American	Argentina	ISSP 2009	0.250326
Canada	South American			No data
Sweden	Chilean	Chile	ISSP 2000–9 (pooled)	0.168185
USA	Mexican	Mexico	ISSP 2000	− 0.2437
Canada	Filipino	Philippines	ISSP 2000–9 (pooled)	0.64454
England	Bangladesh	Bangladesh	SDSA	− 0.01309
Sweden	Horn of Africa	Tanzania (proxy)	Afrobarometer 1	0.589518

## Appendix 4

See Table 6.

**Table 6:** Model estimates restricted to countries for which all three dependent variables are available (Belgium, Canada, England, France, the Netherlands, Sweden)

	(1)	(2)	(3)	(4)	(5)
*Net ethnic gap in student test scores*					
Selective migration index	0.188	0.122	− 0.095	0.035	0.375∼
	(1.47)	(1.63)	(− 0.20)	(0.36)	(1.66)
Same language in origin and destination countries	− 0.030	− 0.053	− 0.050	− 0.035	− 0.044
	(− 0.40)	(− 0.50)	(− 0.46)	(− 0.51)	(− 0.64)
Migrant integration policy (MIPEX/10)		− 0.001	− 0.004		
		(− 0.03)	(− 0.09)		
Selective migration index * (MIPEX/10)			0.029		
			(0.50)		
Tracking index				− 0.158***	− 0.172***
				(− 3.62)	(− 4.28)
Selective migration index * Tracking index					0.327
					(1.61)
Constant	− 0.075	0.052	0.068	− 0.079∼	− 0.086∼
	(− 0.80)	(0.13)	(0.18)	(− 1.67)	(− 1.95)
Variance between countries	0.040***	3.09e−23	1.13e−23	1.11e−24	2.26e−23
	(− 4.54)	(− 0.22)	(− 0.26)	(− 0.24)	(− 0.31)
Variance between ethnic groups within countries	0.043***	0.057***	0.057***	0.047***	0.046***
	(− 14.98)	(− 4.68)	(− 4.74)	(− 5.59)	(− 5.88)
Observations	52	52	52	52	52
*Net ethnic gap in academic versus vocational track*					
Selective migration index	0.739***	0.738***	− 1.637***	0.738***	0.427
	(6.29)	(6.20)	(− 3.57)	(6.20)	(0.51)
Same language in origin and destination countries	− 0.090	− 0.088	− 0.061	− 0.089	− 0.071
	(− 1.09)	(− 1.08)	(− 0.62)	(− 1.08)	(− 0.72)
Migrant integration policy (MIPEX/10)		0.065*	0.049		
		(2.07)	(1.47)		
Selective migration index * (MIPEX/10)			0.297***		
			(5.33)		
Tracking index				− 0.115**	− 0.104*
				(− 3.16)	(− 2.37)
Selective migration index * Tracking index					− 0.318
					(− 0.41)
Constant	0.253**	− 0.167	− 0.0555	0.238***	0.240***
	(2.79)	(− 0.90)	(− 0.29)	(4.48)	(4.13)
Variance between countries	0.017***	0.012***	0.014***	0.006***	0.006***
	(− 6.79)	(− 15.16)	(− 13.95)	(− 10.46)	(− 10.71)
Variance between ethnic groups within countries	0.045***	0.045***	0.040***	0.045***	0.044***
	(− 28.12)	(− 27.90)	(− 18.81)	(− 27.91)	(− 37.52)
Observations	44	44	44	44	44
*Net ethnic gap in completion of upper secondary education*					
Selective migration index	− 0.002	− 0.002	− 0.383	− 0.002	0.143
	(− 0.04)	(− 0.04)	(− 0.83)	(− 0.04)	(0.21)
Same language in origin and destination countries	− 0.101	− 0.101	− 0.097	− 0.101	− 0.110
	(− 1.16)	(− 1.16)	(− 1.08)	(− 1.16)	(− 1.23)
Migrant integration policy (MIPEX/10)		− 0.057	− 0.059		
		(− 0.95)	(− 0.97)		
Selective migration index * (MIPEX/10)			0.049		
			(0.87)		
Tracking index				− 0.204**	− 0.210**
				(− 2.81)	(− 2.79)
Selective migration index * Tracking index					0.146
					(0.23)
Constant	0.089	0.465	0.482	0.023	0.027
	(0.61)	(1.33)	(1.35)	(0.31)	(0.35)
Variance between countries	0.073***	0.070***	0.072***	0.034***	0.036***
	(− 5.46)	(− 4.39)	(− 4.33)	(− 5.34)	(− 5.26)
Variance between ethnic groups within countries	0.033***	0.033***	0.032***	0.033***	0.033***
	(− 22.49)	(− 22.38)	(− 21.41)	(− 22.38)	(− 22.77)
Observations	52	52	52	52	52

*t* statistics in parentheses

∼*p *< 0.10; **p *< 0.05; ***p *< 0.01; ****p *< 0.001

## Appendix 5

See Table 7.

**Table 7:** Model estimates restricted to European countries (excluding Canada and the USA)

	(1)	(2)	(3)	(4)	(5)
*Net ethnic gap in student test scores*					
Selective migration index	0.086	0.101	− 0.246	0.124	− 0.009
	(0.55)	(1.34)	(− 0.70)	(1.38)	(− 0.11)
Same language in origin and destination countries	0.104	0.335**	0.336**	0.187	0.195
	(1.18)	(2.60)	(2.65)	(1.29)	(1.33)
Migrant integration policy (MIPEX/10)		0.170***	0.166***		
		(5.68)	(5.24)		
Selective migration index * (MIPEX/10)			0.049		
			(1.10)		
Tracking index				− 0.175*	− 0.166*
				(− 2.40)	(− 2.31)
Selective migration index * Tracking index					− 0.243∼
					(− 1.94)
Constant	− 0.220*	− 1.344***	− 1.319***	− 0.257∼	− 0.261∼
	(− 2.27)	(− 5.55)	(− 5.19)	(− 1.76)	(− 1.79)
Variance between countries	0.060***	1.61e−20***	1.40e−23	1.34e−23	1.31e−23
	(− 4.84)	(− 4.47)	(− 0.35)	(− 0.23)	(− 0.25)
Variance between ethnic groups within countries	0.067***	0.088***	0.088***	0.126***	0.123***
	(− 14.59)	(− 8.41)	(− 5.48)	(− 3.70)	(− 3.77)
Observations	66	66	66	66	66
*Net ethnic gap in academic versus vocational track*					
Selective migration index	0.727***	0.727***	− 1.071∼	0.727***	0.610***
	(7.12)	(7.09)	(− 1.75)	(7.09)	(5.58)
Same language in origin and destination countries	− 0.055	− 0.054	− 0.044	− 0.055	− 0.050
	(− 0.69)	(− 0.69)	(− 0.53)	(− 0.69)	(− 0.63)
Migrant integration policy (MIPEX/10)		0.083*	0.070∼		
		(2.39)	(1.87)		
Selective migration index * (MIPEX/10)			0.230**		
			(3.08)		
Tracking index				− 0.095***	− 0.088**
				(− 3.55)	(− 3.22)
Selective migration index * Tracking index					− 0.150**
					(− 2.71)
Constant	0.219**	− 0.317	− 0.219	0.222***	0.223***
	(3.00)	(− 1.38)	(− 0.89)	(4.68)	(4.42)
Variance between countries	0.017***	0.010***	0.011***	0.006***	0.006***
	(− 6.95)	(− 18.95)	(− 17.69)	(− 8.14)	(− 8.22)
Variance between ethnic groups within countries	0.047***	0.047***	0.044***	0.047***	0.047***
	(− 22.81)	(− 22.72)	(− 20.12)	(− 22.72)	(− 22.73)
Observations	60	60	60	60	60
*Net ethnic gap in completion of upper secondary education*					
Selective migration index	− 0.028	− 0.028	− 0.583*	− 0.028	0.238
	(− 0.66)	(− 0.66)	(− 2.36)	(− 0.66)	(0.35)
Same language in origin and destination countries	− 0.078	− 0.078	− 0.071	− 0.078	− 0.093
	(− 0.83)	(− 0.83)	(− 0.73)	(− 0.83)	(− 0.96)
Migrant integration policy (MIPEX/10)		− 0.111*	− 0.115*		
		(− 2.12)	(− 2.17)		
Selective migration index * (MIPEX/10)			0.071*		
			(2.35)		
Tracking index				− 0.129*	− 0.133*
				(− 2.18)	(− 2.12)
Selective migration index * Tracking index					0.272
					(0.42)
Constant	− 0.017	0.706∼	0.734∼	− 0.033	− 0.031
	(− 0.15)	(1.86)	(1.91)	(− 0.44)	(− 0.40)
Variance between countries	0.029***	0.014***	0.015***	0.015***	0.015***
	(− 12.41)	(− 15.08)	(− 14.80)	(− 8.11)	(− 7.99)
Variance between ethnic groups within countries	0.029***	0.029***	0.029***	0.029***	0.029***
	(− 37.67)	(− 37.42)	(− 31.83)	(− 37.41)	(− 47.69)
Observations	43	43	43	43	43

*t* statistics in parentheses

∼*p *< 0.10; **p *< 0.05; ***p *< 0.01; ****p *< 0.001

## Footnotes

1 Of course in the USA, forms of within-school tracking exist that are associated with inequalities, but these are typically by subject, and by year, whereas between-school tracking as found in Germany or the Netherlands happens for the full curriculum for a number of years, in separate school buildings and school organizations leading to a different qualification level.

2 One of the dependent variables (enrolment in vocational or academic track within upper secondary education) is conditional on being enrolled in upper secondary education.

3 We used fathers’ education as their educational attainment is arguably a better measure of migration motivation than mothers’ education, for most of the developing countries in the generations studied.

4 For all origin countries, we take males born between 1940 and 1964 as the comparison group. As the origin country, we of course prefer to use the most common origin country of an ethnic group in the destination country. The selection of the origin country, however, also depends on availability of survey data. In a few cases, a proxy having a similar level of human development, as measured by the United Nation’s Human Development Index, was used because no data were available on that particular country: the Dominican Republic as proxy for Caribbean migrants in France, and Tanzania for migrants from Africa’s Horn in Sweden. See "Appendix 3" for details on the data used.

5 www.mipex.eu/methodology (last accessed June 26, 2015).

6 Although the MIPEX index is available since 2007, we had to use the 2010 index because otherwise not all countries could be used in our analyses.

7 We also examined the multiculturalism index of 2000, an index created by Banting and Kymlicka (2006) to measure the degree to which policies toward immigrants are multicultural in the sense that they “extend some level of public recognition and support for ethnocultural minorities to maintain and express their distinct identities and practices” (Banting and Kymlicka 2006). Results with this measurement are very similar to the ones presented here. These results are available upon request.

8 A replication on only European countries (Table 7 of Appendix) showed a negative association between migrant integration policies and the slope for migration background, indicating stronger disadvantages in countries with more migrant-friendly policies.
